# Arm hand skilled performance in cerebral palsy: activity preferences and their movement components

**DOI:** 10.1186/1471-2377-14-52

**Published:** 2014-03-19

**Authors:** Ryanne JM Lemmens, Yvonne JM Janssen-Potten, Annick AA Timmermans, Anke Defesche, Rob JEM Smeets, Henk AM Seelen

**Affiliations:** 1 Research School CAPHRI, Department of Rehabilitation Medicine, Maastricht University, Maastricht, The Netherlands; 2 Adelante, Centre of Expertise in Rehabilitation and Audiology, Hoensbroek, The Netherlands; 3 BIOMED Biomedical Research Institute, Hasselt University, Diepenbeek, Belgium; 4 Adelante Rehabilitation Centre, Valkenburg, The Netherlands

**Keywords:** Cerebral Palsy, Children, Adolescents, Activities of daily living, Upper extremity, Treatment goals, Training preferences, Canadian Occupational Performance Measure, Movement components, Rehabilitation

## Abstract

**Background:**

Assessment of arm-hand use is very important in children with cerebral palsy (CP) who encounter arm-hand problems. To determine validity and reliability of new instruments to assess actual performance, a set of standardized test situations including activities of daily living (ADL) is required. This study gives information with which such a set for upper extremity skill research may be fine-tuned, relative to a specific research question. Aim of this study is to a) identify upper extremity related ADL children with CP want to improve on, b) determine the 10 most preferred goals of children with CP, and c) identify movement components of all goals identified.

**Method:**

The Canadian Occupational Performance Measure was used to identify upper extremity-related ADL preferences (goals) of 53 children with CP encountering arm-hand problems (mean age 9 ± 4.5 year). Goals were ranked based on importance attributed to each goal and the number of times a goal was mentioned, resulting in a gross list with goals. Additionally, two studies were performed, i.e. study A to determine the 10 most preferred goals for 3 age groups (2.5-5 years; 6-11 years, 12-19 years), based on the total preference score, and study B to identify movement components, like reaching and grasping, of all goals identified for both the leading and the assisting arm-hand.

**Results:**

Seventy-two goals were identified. The 10 most preferred goals differed with age, changing from dressing and leisure-related goals in the youngest children to goals regarding personal care and eating for children aged 6-11 years. The oldest children preferred goals regarding eating, personal care and computer use. The movement components ‘positioning’, ‘reach’, ‘grasp’, and ‘hold’ were present in most tasks. ‘Manipulating’ was more important for the leading arm-hand, whereas ‘fixating’ was more important for the assisting arm-hand.

**Conclusion:**

This study gave insight into the preferences regarding ADL children with CP would like to improve on, and the movement components characterizing these activities. This information can be used to create a set of standardized test situations, which can be used to assess the validity and reliability of new measurement instruments to gauge actual arm-hand skilled performance.

## Background

Cerebral Palsy (CP) is a group of permanent, but not unchanging, disorders of movement and/or posture and of motor function, which are due to a non-progressive interference, lesion, or abnormality of the developing/immature brain [1]. About 60% of the children with CP has problems with their arm-hand (i.e. Manual Ability Classification System (MACS) > 1) [2]. Arm-hand skilled performance, i.e. the use of the arm and hand during activities of daily living (ADL), is important to live independently.

In rehabilitation, assessment of arm-hand performance is important to monitor therapy progress and determine the effectiveness of therapies. Whereas many instruments exist to assess arm-hand functioning on the level of capacity or perceived performance, instruments to (validly and reliably) assess actual performance, i.e. measuring what a patient actually does with his/her arm-hand in daily life, are still lacking [3]. For example, instruments containing multiple sensors may measure skill performance objectively [4,5]. To test the validity and reliability of such new instruments, a set of standardized test situations, featuring arm-hand activities mimicking ADL, is essential. These activities should be representative for activities children with CP want to improve on. In the present paper, activities children with CP want to improve on will be referred to as ‘goals’.

Nieuwenhuijsen showed that, in young adults with CP, problems in ADL were most prevalent in recreation, preparing meals, housework and dressing [6]. Livingston concluded that the three most frequently identified issues regarding participation in adolescents with CP were related to ‘paid work’, meal ‘preparation’ and ‘socializing with friends’ [7]. Majnemer studied the leisure activity preferences for 6–12 year old children with CP [8], and identified that social and recreational activities were most preferred, whereas self-improvement activities (as writing) were least preferred. Nevertheless, currently, for children with CP in any age group, no clear overview exists of goals with regard to upper extremity ADL.

In the present study, goals regarding upper extremity related ADL of children with CP of all ages were inventoried. Once these goals are known, it is possible to create a set of standardised test situations containing well-described arm-hand activities, usable to validate and test reliability of new instruments, and, eventually, usable to evaluate arm-hand performance. This test set containing arm-hand activities should meet several requirements, that is: 1) include *standardized* activities, to be able to test the reproducibility of instruments; 2) be *valid*, i.e. including activities important for children with CP; 3) be *compact,* since it is impossible to measure all activities; 4) include activities *covering all movement components* (like reaching, grasping and manipulating) of arm-hand skilled performance [9]; 5) be *sensitive* to detect differences between subpopulations of CP and detect changes over time, during and after training. Activities should be chosen in such a way that the whole spectrum of movement components is adequately represented, yet the set of standardised test situations is kept concise.

This study is not aimed at providing an ultimate set of standardized test situations. It rather provides the reader with information with which a set of standardized test situations for upper extremity skill research may be fine-tuned, relative to a specific research question. The aim of this study is to a) identify upper extremity related ADL children with CP want to improve on, b) determine the 10 most preferred goals of children with CP in three different age groups, and c) identify movement components of all goals identified.

## Methods

### Study design and participants

In this cross-sectional study, a convenience sample of 53 children with CP was included. The older children participated in a constraint-induced movement therapy (CIMT) program and the younger children participated in an effect study (BOBIVA, ISRCTN69541857, approved by the MEC of Atrium Medical Centre Heerlen, The Netherlands). Children were recruited in Adelante Rehabilitation Centre, Valkenburg, The Netherlands. Children and/or parent(s) signed informed consent before the start of the study. Inclusion criteria were: 1) Age 2.5–20 years (2.5-12 for BOBIVA and 11–20 for CIMT); 2) diagnosed with unilateral spastic CP [10]; 3) spastic hemiparesis or extreme asymmetric diplegia according to the Hagberg criteria; 4) hand function impairment Zancolli grade I with evident problems in thumb extension and supination, or Zancolli grade IIA or IIB [11]; 5) mentally able to comprehend and perform tasks, as judged by the rehabilitation physician; 6) child and its parent(s) should comprehend and speak Dutch. Exclusion criteria were: 1) severe structural contractures of the muscles of the upper extremity, i.e. a) passive elbow extension less than 160 degrees, b) supination less than 30 degrees from neutral position, c) wrist dorsal flexion less than 20 degrees (children aged 2.5-6 years) or less than 45 degrees (children aged 7–18 years), 2) severe impairment of hand function (Zancolli III); 3) hand surgery or phenolisation or Botulinum Toxin-A (btA) injections in the arm less than nine months ago; 4) children who cannot bare touching the affected arm and hand.

### Procedure and data analysis

#### Identification of goals and preference scores

The MACS [12] and Gross Motor Function Classification System (GMFCS) [13] scores were retrieved from the participants’ medical files. The Canadian Occupational Performance Measure (COPM) [14] was used to identify the individual activities children wanted to improve on, referred to as ‘goals’. The COPM was administered, by trained and certified occupational therapists, prior to the start of each of the studies from which patients were identified. Depending on the age of the child and whether the child had difficulties with verbal and/or gestural expression, the goals were set by the child itself (cognitive age >6 years), or with help of the parent(s) and therapist, and goals were ranked based on importance. The goals had to consist of activities in which at least one upper extremity is involved. A preferencse score was given to the goals based on their ranking, i.e. the most preferred goals receiving the highest ranking [15]. As the number of goals identified differed from 1 to 6, a score of 6 was given to the most important goal, 5 for the next goal, and so on. If a goal consisted of 2 different activities, these activities were analysed as separate goals with the same preference score. For example, ‘putting on my sweater and trousers’ was subdivided into: ‘putting on one’s sweater’ and ‘putting on the trousers’. A gross list of goals was generated, including all goals of all children. Similar goals (i.e. the same goal mentioned by multiple children) were clustered.

**Figure 1:**
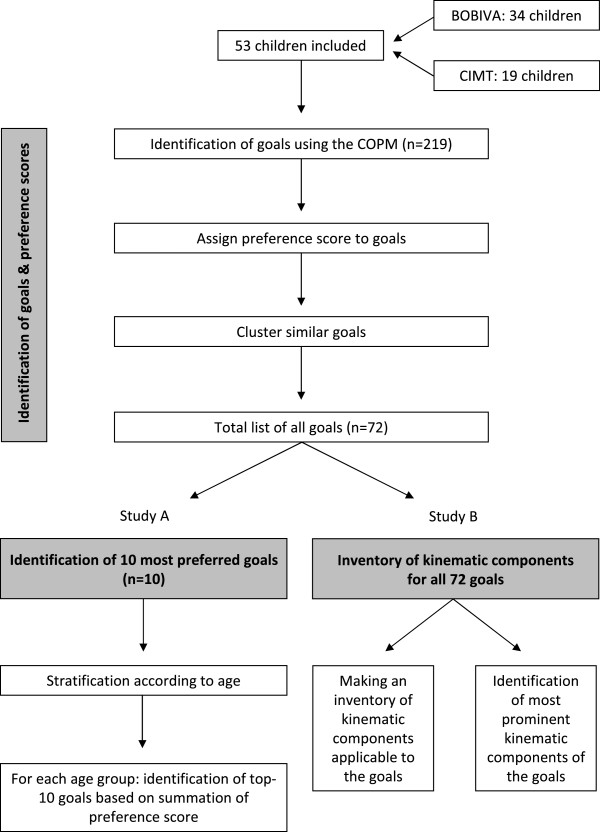
Process of goal identification, as well as inventory of movement components and most preferred goals.

After the identification of the goals, two studies were performed, i.e. study A to determine the 10 most preferred goals of children with CP in three different age groups and study B to identify movement components of all goals identified. Figure 1 depicts the process of i) goal identification, ii) inventory of most preferred goals and iii) inventory of movement components.

#### Identification of 10 most preferred goals (study A)

The participant’s preference score was used to identify the 10 goals most preferred, for each of three age groups (2.5-5 years, 6–11 years, 12–19 years). This stratification by age was made because the activities children perform vary with age and sensorimotor development [16]. Around the age of 12, motor skills, like reaching and grasping, reach mature levels [17,18]. During adolescence, changes in interest of children lead to different activity preferences.

Per age group, for each goal, the number of children that identified that goal was counted. A total preference score per goal was calculated by summing the individual preference scores. From the total preference score, the top-10 most preferred goals were identified for each age group.

#### Inventory of movement components (study B)

The gross list of 72 goals was analyzed, based on their movement components. Ten movement components were defined a-priori (Table 1)*,* based on a procedure described by Timmermans et al. [15].

**Table 1:** Movement components and their definition

**Movement component**	**Definition**
Positioning the upper extremity	Maintaining a fixed position of the shoulder, arm and/or hand in space.
Reach	Intentional movement of the arm towards an object
Grasp	To make a motion of seizing, snatching or clutching
Hold	Keep an object in a fixed position in the hand without external support
Release	To free an object from grip
Manipulate	To skilfully control the position of an object using the fingers
Push/Pull/Shove	To apply force against an object with the intention to move or stabilize
Displace/Lift	Moving an object without the object being in contact with a surface in the environment
Fixate	To stabilize an object against a surface
Other	Other movement components not covered by the abovementioned movement components

For each goal, it was inventoried which movement components are present in the goal and which components are most prominent. Both inventories were done separately for the leading arm-hand and the assisting arm-hand. The leading arm-hand (i.e. the non-impaired arm-hand in children with CP and the dominant arm-hand in healthy children) is preferred for precision tasks, whereas the assisting arm-hand (the impaired arm-hand in children with CP and the non-dominant arm-hand in healthy children) has a more complementary, holding and stabilizing role [19]. For each goal, it was specified a-priori which hand is the leading hand and which hand is the assisting hand. For instance for the goal ‘playing tennis’ the leading hand would hold the racket and the assisting hand would manipulate the ball (see Additional file 1).

Four experts in the field of movement sciences and arm-hand rehabilitation evaluated the goals to identify the movement components. The list of goals was randomly divided in two parts. For each part, two experts (part 1: YJ&HS; part 2: AT&RL) identified the movement components present, and the most prominent movement component(s) for both the leading and the assisting arm-hand.

The definitions in Table 1 were used as a guideline to attribute movement components to goals. In addition, a-priori agreements were made to ensure all experts had the same point of view. Because arm-hand skilled performance varies with disease severity, the execution of the activity by a healthy person served as a reference. Additionally, the assumed starting position for the execution of the goals was defined as ‘sitting with hands in lap’, or ‘standing with hands and arms along the body’.

One of the prerequisites for the inventory of movement components present in a goal was that a component should factually constitute to the achievement of that goal. For the inventory of the most prominent component(s), it was checked which movement component(s) characterize(s) the goal *best.*

The degree of agreement was calculated as the ratio (in %) of ‘the total number of components the reviewers agreed on for all goals’ and ‘the total number of components for all goals’. In case of disagreement, the experts discussed the case until consensus was reached.

## Results

### Demographic and medical variables

Participants’ characteristics are displayed in Table 2. Male/female ratio was 36/17. The socio-economic background of the children included in this study did not differ from the general population.

**Table 2:** Patient characteristics

		**Total**	**Per Age group**		
		**2.5-19 years**	**2.5-5 years**	**6-11 years**	**12-19 years**
**Number of children**		53	13	22	18
**Mean age (sd)**		9 (4.5)	4 (1.0)	8 (2.0)	14 (2.4)
**Hemiparesis**	**Left**	23	5	10	8
(Number of patients)	**Right**	30	8	12	10
**MACS**	**I.**	20	5	7	8
(Number of children)	**II.**	19	5	10	4
	**III.**	14	3	5	6
	**IV.**	0	0	0	0
	**V.**	0	0	0	0
**GMFCS**	**I.**	48	10	21	17
(Number of children)	**II.**	5	3	1	1
	**III.**	0	0	0	0
	**IV.**	0	0	0	0
	**V.**	0	0	0	0

MACS = Manual Ability Classification System; GMFCS = Gross Motor Function Classification System.

### Inventory of goals

In total, 219 goals were identified. After clustering similar goals, a list of 72 goals remained.

### Identification of 10 most preferred goals (study A)

**Figure 2:**
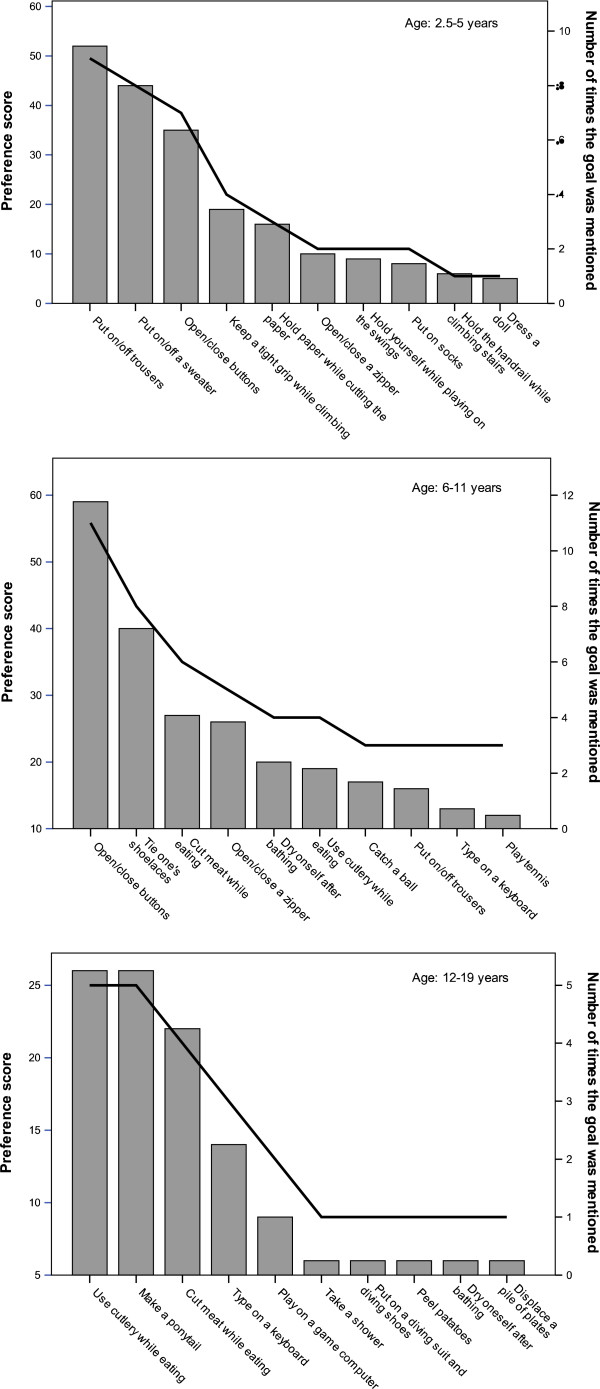
**Top-10 most preferred training goals for children aged between 2.5-5 year (top panel), 6–11 year (middle panel) and 12–19 year (bottom panel) as determined by the COPM and the preference score.** Bars indicate to total preference score (left y-axis) and the line indicates the number of times the goal was mentioned (right y-axis).

Figure 2 shows the 10 goals with the highest preference score for each age group separately. In all age groups, all goals in the top-10 consisted of bimanual activities, except the goal ‘hold the handrail while climbing stairs’ in the youngest children.

In the youngest children, most goals in the top-10 concerned dressing or leisure activities. In children aged 6–11 years, dressing was still preferred, but eating, personal care and leisure goals were also rated as highly important. The oldest children preferred to improve on tasks regarding eating as well as personal care and leisure. Goals concerning leisure were present in all age groups, but the goals changed with age, i.e. from climbing and playing on the swings in the youngest group to activities with a ball in the age group 6–11 years and using the computer in the oldest age group. Additional file 1 contains a list of all goals for the three age groups separately (Additional file 1: Table A1-3).

### Inventory of the movement components (study B)

For each of the 72 goals identified, the movement components present and the most prominent movement component(s) were inventoried. The goals ‘work in the garden’ and ‘rig up a sailing boat’ were too vaguely described and were therefore excluded for this part of the analysis (thus 70 goals were further analysed). For the movement components present, upon initial evaluation (before 100% agreement was achieved) the mean degree of agreement between experts was 80.4% for the leading arm-hand and 75.9% for the assisting arm-hand. For the most prominent component(s), the mean degree of agreement was 82.7% for the leading arm-hand and 81.3% for the assisting arm-hand.

**Figure 3:**
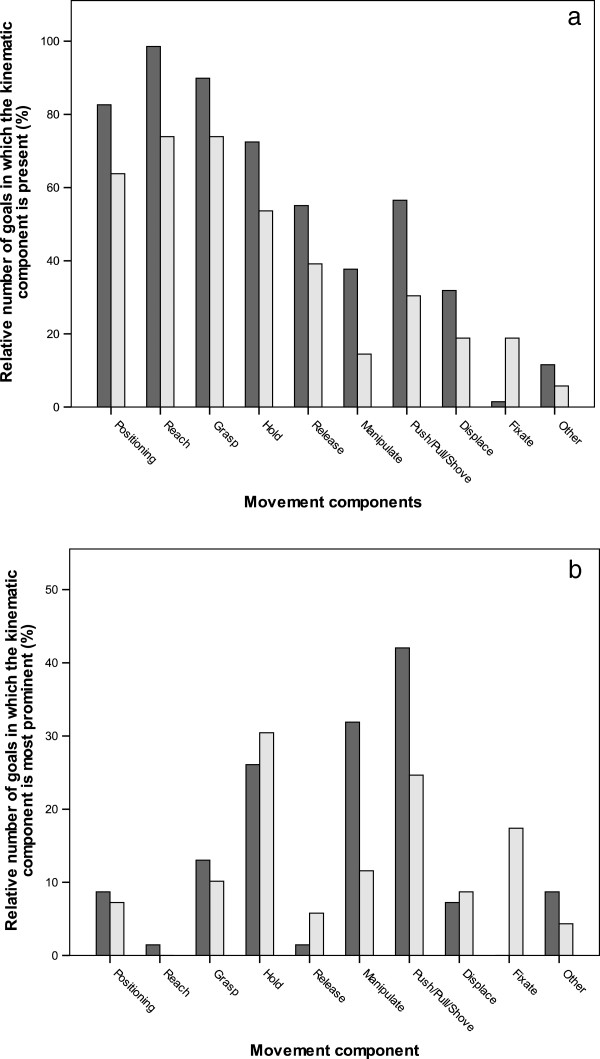
**Inventory of movement components: a) for each movement component, the percentage of goals in which the movement component was present; b) the percentage of goals in which the movement component has been identified as the most prominent movement component.** Dark grey bars represent the leading arm-hand and light grey bars represent the assisting arm-hand.

Figure 3a shows, per movement component, the percentage of goals in which this component was present. The components ‘positioning’, ‘reach’, ‘grasp‘ and ‘hold’ were present in over 60% of all goals. Furthermore, for the leading arm-hand, relative to assisting arm-hand, all components except ‘fixate’, were more often present in a goal.

Figure 3b shows the percentage of goals in which a specific movement component is identified as being most prominent. For the leading arm-hand, the components ‘hold’, ‘manipulate’ and ‘push/pull/shove’ were most frequently assigned as most important. For the assisting arm-hand, the components ‘hold’, ‘push/pull/shove’ and ‘fixate’ were most frequently assigned as the most important. The most obvious difference between the leading and the assisting arm-hand is the component ‘fixate’ which was much more often assigned for the assisting arm-hand, and the components ‘manipulate’ and ‘push/pull/shove’, which were more frequently assigned for the leading arm-hand.

Table 3 depicts the most prominent movement components of the goals among the top-10 most preferred goals. For the leading arm-hand, manipulate and push/pull/shove were in indicated most frequently, whereas for the assisting arm-hand, the components were distributed more evenly. The component reach was not indicated as most prominent for both the leading and the assisting arm-hand. The components release and fixate were not indicated as most prominent for the leading arm-hand in any of these goals. Additional file 2 shows the most prominent movement components of all goals 70 goals.

**Table 3:** Overview of the most prominent movement components for the goals listed in the top-10 most preferred goals pooled for all three age groups

	**Leading arm-hand**										**Assisting arm-hand**									
	**Indicated as most prominent**										**Indicated as most prominent**									
	**P**	**Rea**	**G**	**H**	**R**	**M**	**P/P/S**	**D**	**F**	**O**	**P**	**Rea**	**G**	**H**	**R**	**M**	**P/P/S**	**D**	**F**	**O**
Open/Close buttons						**X**													**X**	
Put on/off trousers						**X**							**X**							
Put on/off a sweater							**X**										**X**			
Tie one’s shoelaces						**X**										**X**				
Cut meat while eating							**X**												**X**	
Open/Close a zipper						**X**	**X**												**X**	
Use cutlery while eating							**X**							**X**				**X**		
Make a ponytail						**X**										**X**				
Dry oneself after bathing							**X**										**X**			
Keep a tight grip while climbing			**X**				**X**						**X**				**X**			
Catch a ball				**X**										**X**						
Type on a keyboard							**X**										**X**			
Hold paper while cutting the paper	**X**					**X**					**X**								**X**	
Play tennis	**X**			**X**											**X**					
Hold yourself while playing on the swings							**X**										**X**			
Play on a game computer						**X**								**X**		**X**				
Put on socks							**X**										**X**			
Hold the handrail while climbing stairs	**-**	**-**	**-**	**-**	**-**	**-**	**-**	**-**	**-**	**-**			**X**				**X**			
Displace a pile of plates				**X**				**X**						**X**				**X**		
Peel potatoes							**X**							**X**		**X**				
Put on a diving suit and diving shoes							**X**										**X**			
Take a shower (washing one’s armpit and back)							**X**										**X**			
Dress a doll						**X**							**X**		**X**					

- = not applicable.

P = positioning; Rea = reach; G = grasp; H = hold; R = release; M = manipulate; P/P/S = push/pull/shove; D = displace; F = fixate; O = other.

## Discussion

This study provides the reader with information with which a set of standardized test situations for upper extremity skill research may be fine-tuned, relative to a specific research question. The aim of this study is to a) identify upper extremity related ADL children with CP want to improve on, b) determine the 10 most preferred goals of children with CP in three different age groups, and c) identify movement components of all goals identified.

### Main findings regarding most preferred goals

The 10 most preferred goals differed with age. It is possible to make a distinction between primary individual needs, including goals regarding dressing and eating, and social demands, including leisure activities. In all age groups, the top-10 goals consisted of both primary needs and social demands. The youngest children preferred mainly dressing and leisure activities. While these activities are still important for children aged 6–11 years, they rated skills regarding eating and personal care also as being important. For the oldest children, skills regarding eating were most important, followed by personal care and using the computer. This age-related change in preferences is caused by children developing rapidly and learning new motor skills. Majnemer, similarly, showed age-related differences in leisure activity preferences of children with CP aged 6–12 [8]. Goals regarding leisure/playing were also present in our top-10 but these were not ranked very high. This might be caused by the fact that there is a lot of variation in playing activities, decreasing the chance that children named the same leisure activities as being important.

Comparing the goals of children with CP with skills of typically developing children, it appears that the former encounter more difficulties in performing ADL. For example, dressing is the most important goal in children with CP aged 6–11 years, whereas most typically developing children have mastered this skill at that age [20].

When comparing goals identified in the present study with those reported in literature, both similarities and differences are observed. Donkervoort found that young adults with CP encounter problems with self-care and nutrition [21], two domains also scoring high in the present study. Nieuwenhuijsen investigated the experienced problems in young adults with CP and found that, in contrast to the present study, housework was rated as being important. This difference can be explained by the age difference, though relatively small, between populations. Young adults are likely to live more independently and more likely to experience problems in the performance of housework activities.

In all age groups multiple children wanted to improve on the same goal. For instance, 69% of the children aged 2.5-5 years wanted to improve on the goal ‘put on trousers’, a skill that is typically under development in this age group.

Nearly all goals children set were bimanual activities. It must be taken into account that the question for setting goals was: “on which task do you want to improve using your affected arm-hand?”. Since children with CP use their affected arm-hand as the assisting arm-hand, it is logical that the goals for improvement are bimanual activities in which the affected arm-hand is used as support.

Timmermans et al. investigated training preferences of stroke patients [15]. Interestingly, several activities children with CP rated as important are also preferred by stroke patients. The 10 goals with the highest preference score for stroke patients included, amongst others, eating, using the computer, dressing and self-care, all of which were rated as important in the current study. Similarity between preferred goals across patient populations may offer opportunities to apply similar therapies and measurement instruments.

### Main findings regarding the movement components of the goals

In total, 72 goals were identified. All goals consisted of multiple movement components. The inventory of movement components was not restricted to the top-10 most preferred goals but to all goals identified, because it is highly likely that not all movement components will be covered by the top-10 most preferred goals. In line with Timmermans et al., who inventoried the movement components of training preferences of stroke patients [15], the components ‘positioning’, ‘reach’, ‘grasp’ and ‘hold’ were present in most activities. These components are required when handling objects, necessary in the majority of the goals. ‘Manipulate’, more frequently assigned to the leading arm-hand, is often associated with fine motor skills. ‘Fixate’ requires less fine motor skills and may therefore, in bimanual tasks, predominantly be performed by the assisting arm-hand. ‘Hold’, ‘push/pull/shove’, ‘manipulate’ (only for the leading arm-hand) and ‘fixate’ (only for the assisting arm-hand) were most frequently rated as most prominent movement component.

The components ‘manipulate’, ‘fixate’ and ‘push/pull/shove’ are quit specific and characterize a goal. They are not present in many goals, but, when present, they are frequently rated as most prominent. The components ‘reach’ and ‘release’, present in many goals, though not often indicated as most prominent. To be able to grasp an object, reaching and releasing are required.

### Implication for the development of the set standardized test situations

In order to be able to test the validity and reliability of newly developed instruments to assess actual performance, a set of standardized test situations is needed. This study is not aimed at providing an ultimate set of standardized test situations. It rather provides the reader with information with which a set of standardized test situations for upper extremity skill research may be fine-tuned, relative to a specific research question.

In selecting activities for a valid test set, the following points should be taken into account:

– All movement components of arm-hand skilled performance should be covered.

– For every movement component, an activity should be included in which that component is identified as being most prominent, ensuring that all components are represented adequately in the test set.

– Activities should be included which are important for children with CP.

When selecting goals covering all movement components, it may be necessary to include goals not listed in the top-10 most preferred goals. For instance, the movement component ‘reach’ is only indicated as most prominent in the goal ‘shaking hands’, not listed in the top-10. Furthermore, it must be considered whether the selected goals are relevant for the participants. Goals less relevant for a specific patient may be interchanged with other goals containing the same movement components. For example, the goal ‘make a ponytail’, probably not performed by boys, may be replaced by the goal ‘tying one’s shoelaces’ containing the same most prominent components.

All movement components (except ‘fixate’ for the leading arm-hand and ‘reach’ for the assisting arm-hand) are characterized as being most important at least once within the following set of goals: ‘use cutlery while eating’, ‘catch a ball’, ‘hold paper while cutting paper’, ‘put on socks’, ‘displace a pile of plates’, ‘shake hands’ and ‘use modelling clay’. The combination of these activities may be used to form a test set, comprehensively covering most movement components. However, the exact choice of activities may vary depending on the research question of a study and the aim of using such test set.

### Considerations

This study has some limitations. The choice of goals children wanted to improve on might have been influenced by the socio-economic background of the children and their family. It was not the scope of this paper to investigate this influence. And because the socio-economic background of the children included in this study did not differ from the general population, no large effects were expected. Disease severity is another factor that might influence the choice of goals of the children. In this study, only children with a MACS score of I, II or III and a GMFCS score of I or II were included. Children with severe structural contractures and severe impairment of the hand were excluded. This must be kept in mind when interpreting these data, together with the relatively low sample size.

The inventory of movement components by multiple experts is a point of discussion. An activity can be performed in several ways, influencing the choice of components. However, in the approach used in this study, a-priori agreements were defined to minimize these differences. The experts independently identified these movement components with a mean degree of agreement of 80%.

## Conclusions

This study gave insight into the preferences regarding ADL children with CP (aged between 2.5-19 years) would like to improve on. Together with the information about the movement components characterizing these activities, this information can be used to create a set of standardized test situations. Such a test-set can be used for multiple purposes, depending on one’s research question. One of the situations in which this set of standardized activities will be used is to validate and test the reproducibility of a new measurement instrument to assess actual arm-hand skilled performance.

## Abbreviations

ADL: Activities of daily living; CP: Cerebral palsy; MACS: Manual ability classification system; CIMT: Constrained induced movement therapy; btA: Botulinum Toxin-A; GMFCS: Gross motor function classification system; COPM: Canadian occupational performance measure.

## Competing interests

The authors declare that they have no competing interests.

## Authors’ contributions

RL participated in the analysis of the data, participated in interpreting the results and drafted the manuscript. YJ participated in the analysis of the data, participated in interpreting the results and revised the manuscript critically. AT participated in the analysis of the data, participated in interpreting the results and revised the manuscript critically. AD carried out the data collection. RS participated in interpreting the results and revised the manuscript critically. HS participated in the analysis of the data, participated in interpreting the results and revised the manuscript critically. All authors read and approved the final manuscript.

## Pre-publication history

The pre-publication history for this paper can be accessed here:

http://www.biomedcentral.com/1471-2377/14/52/prepub

## Supplementary Material

Additional file 1 For the goals in which the performance of the leading arm-hand (LH) differs from the performance of the assisting arm-hand (AH), the role of each hand in the activity described. Click here for file

Additional file 2: Table A2 Overview of the most prominent movement components for the goals identified, pooled for all three age groups. Click here for file
