# Supplementary material for: Arm hand skilled performance in cerebral palsy: activity preferences and their movement components
Source: BMC Neurol. 2014 Mar 19;14:52. doi: 10.1186/1471-2377-14-52 (PMC4000003; doi:10.1186/1471-2377-14-52)
Supplement: Additional file 1 — For the goals in which the performance of the leading arm-hand (LH) differs from the performance of the assisting arm-hand (AH), the role of each hand in the activity described. [file 1471-2377-14-52-S1.doc]

***Additional File 1***

For the goals in which the performance of the leading arm-hand (LH) differs from the performance of the assisting arm-hand (AH), the role of each hand in the activity described.

Table A1: Goals identified with the COPM in children with CP, aged between 2.5 and 5 years

| Rank | Goal | Total preference score | Number of times the goal was mentioned |
| --- | --- | --- | --- |
| 1 | Put on/off trousers | 52 | 9 |
| 2 | Put on/off a sweater | 44 | 8 |
| 3 | Open/close buttons  *LH: Holds the part with the buttonhole*  *AH: Holds the side with the button* | 35 | 7 |
| 4 | Keep a tight grip while climbing | 19 | 4 |
| 5 | Hold paper while cutting the paper  *LH: Uses the scissors*  *AH: Holds the paper* | 16 | 3 |
| 6 | Open/close a zipper  *LH: Manipulates the zipper*  *AH: Fixates the bottom of the coat tail* | 10 | 2 |
| 7 | Hold yourself while playing on the swings | 9 | 2 |
| 8 | Put on socks | 8 | 2 |
| 9 | Hold the handrail while climbing stairs  *LH: Not used*  *AH: Holds the handrail* | 6 | 1 |
| 10 | Dress a doll  *LH: Handlesclothes and fine manipulation*  *AH: Holds the doll and handles clothes* | 5 | 1 |
| 11 | Hold a plate, mug, jar or bowl | 5 | 1 |
| 12 | Put on a coat | 5 | 1 |
| 13 | Tie one’s shoelaces | 5 | 1 |
| 14 | Use cutlery while eating | 4 | 1 |
| 15 | Put on shoes (without shoelaces) | 4 | 1 |
| 16 | Play with Duplo and K'nex | 4 | 1 |
| 17 | Hold toys | 4 | 1 |
| 18 | Hold the handlebars of a bicycle | 4 | 1 |

Table A2: Goals identified with the COPM in children with CP, aged between 6 and 11 years

| Rank | Goal | Total preference score | Number of times the goal was mentioned |
| --- | --- | --- | --- |
| 1 | Open/close buttons  *LH: Holds the part with the buttonhole*  *AH: Holds the side with the button* | 59 | 11 |
| 2 | Tie one’s shoelaces | 40 | 8 |
| 3 | Cut meat while eating  *LH: Uses the knife*  *AH: Uses the fork* | 27 | 6 |
| 4 | Open/close a zipper  *LH: Manipulates the zipper*  *AH: Fixates the bottom of the coat tail* | 26 | 5 |
| 5 | Dry oneself after bathing | 20 | 4 |
| 6 | Use cutlery while eating | 19 | 4 |
| 7 | Catch a ball | 17 | 3 |
| 8 | Put on/off trousers | 16 | 3 |
| 9 | Type on a keyboard | 13 | 3 |
| 10 | Play tennis  *LH: Handles the racket*  *AH: Handles the ball* | 12 | 3 |
| 11 | Make a sandwich  *LH: Uses the knife*  *AH: Fixates the sandwich* | 12 | 2 |
| 12 | Cut bread while eating  *LH: Uses the knife*  *AH: Holds the bread* | 12 | 2 |
| 13 | Displace boxes with toys | 11 | 2 |
| 14 | Play on a game computer | 10 | 2 |
| 15 | Hold the handlebars of a bicycle | 9 | 2 |
| 16 | Hanging on the horizontal bar or swinging on the rings | 7 | 2 |
| 17 | Put on/off a sweater | 6 | 1 |
| 19 | Put on socks | 5 | 1 |
| 20 | Comb one’s hair, make a ponytail | 5 | 1 |
| 21 | Lift a bicycle | 5 | 1 |
| 22 | Making a knot to close a balloon | 5 | 1 |
| 23 | Hold paper while cutting the paper  *LH: Uses the scissors*  *AH: Holds the paper* | 5 | 1 |
| 24 | Disassemble Lego toy building blocks | 5 | 1 |
| 25 | Rig up a sailing boat | 5 | 1 |
| 26 | Put on shin guards  *LH: Fasten the shin guards*  *AH: Fixates the shin guards on the leg* | 4 | 1 |
| 27 | Dress a small toy doll  *LH: Handles the clothes*  *AH: Holds the doll* | 4 | 1 |
| 28 | Play rope-skipping | 4 | 1 |
| 29 | Work in the garden | 4 | 1 |
| 30 | Holding weights with two hands | 4 | 1 |
| 31 | Handle a hockey stick | 4 | 1 |
| 32 | Pulling on sailing ropes | 4 | 1 |
| 33 | Use keys  *LH: Uses the keys*  *AH: Not used* | 3 | 1 |
| 34 | Put on a watch  *LH: Handles the watch*  *AH: Not used* | 2 | 1 |
| 35 | Shake hands with someone  *LH: Shake hands*  *AH: Not used* | 2 | 1 |
| 36 | Play the piano | 2 | 1 |
| 37 | Use the handbrakes while bicycling | 1 | 1 |

Table A3: Goals identified with the COPM in children with CP, aged between 12 and 19 years

| Rank | Goal | Total preference score | Number of times the goal was mentioned |
| --- | --- | --- | --- |
| 1 | Use cutlery while eating | 26 | 15 |
| 2 | Comb one’s hair, make a ponytail | 26 | 5 |
| 3 | Cut meat while eating  *LH: Uses the knife*  *AH: Uses the fork* | 22 | 4 |
| 4 | Type on a keyboard | 14 | 3 |
| 5 | Play on a game computer | 9 | 2 |
| 6 | Displace a pile of plates | 6 | 1 |
| 7 | Peel potatoes  *LH: Uses the knife*  *AH: Holds the potato* | 6 | 1 |
| 8 | Put on a diving suit and diving shoes | 6 | 1 |
| 9 | Dry oneself after bathing | 6 | 1 |
| 10 | Take a shower | 6 | 1 |
| 11 | Shake hands with someone  *LH: Shake hands*  *AH: Not used* | 6 | 1 |
| 12 | Use keys  *LH: Uses the keys*  *AH: Not used* | 6 | 1 |
| 13 | Cut a paper/cardboard template  *LH: Uses the knife*  *AH: Fixates paper/cardboard* | 6 | 1 |
| 14 | Play tennis  *LH: Handles the racket*  *AH: Handles the ball* | 6 | 1 |
| 15 | Operating machines  *LH: Pushes buttons*  *AH: Not used* | 6 | 1 |
| 16 | Put screws in  *LH: Handles the screwdriver*  *AH: Holds the screw* | 6 | 1 |
| 17 | Open a bottle  *LH: Handles the cap*  *AH: Holds the bottle* | 5 | 1 |
| 18 | Pour a drink  *LH: Handles the bottle/pitcher*  *AH: Not used* | 5 | 1 |
| 19 | Put on a glove  *L: Handles glove*  *A: Positions hand to put glove on* | 5 | 1 |
| 20 | Fasten a bracelet  *LH: Handles the bracelet*  *AH: Not used* | 5 | 1 |
| 21 | Pick up a tennis ball | 9 | 2 |
| 22 | Displace more than 3 glasses at the same time | 5 | 1 |
| 23 | Wood crafting  *LH: Handles the tools*  *AH: Fixates the wood* | 5 | 1 |
| 24 | Keep a tight grip while climbing | 5 | 1 |
| 25 | Displace a kettle and drain water for boiled potatoes | 4 | 1 |
| 26 | Cut vegetables/fruit  *LH: Uses the knife*  *AH: Fixates the vegetables/fruit* | 4 | 1 |
| 27 | Open/close buttons  *LH: Holds the part with the buttonhole*  *AH: Holds the side with the button* | 4 | 1 |
| 28 | Tie one’s shoelaces | 4 | 1 |
| 29 | Catch a ball | 4 | 1 |
| 30 | Play the guitar  *LH: Plucks the strings*  *AH: Holds the guitar* | 4 | 1 |
| 31 | Grasp and release objects  *LH: Grasps the object*  *AH: Not used* | 4 | 1 |
| 32 | Do a handstand | 4 | 1 |
| 33 | Use modelling clay | 4 | 1 |
| 34 | Making a knot to close a balloon | 4 | 1 |
| 35 | Use a mechanic mouse  *LH: Handles the mechanic mouse*  *AH: Not used* | 4 | 1 |
| 36 | Pack a back | 4 | 1 |
| 37 | Lift a pet | 4 | 1 |
| 38 | Hold a jar while opening it  *LH: Opens the jar*  *AH: Holds the jar* | 3 | 1 |
| 39 | Play badminton  *LH: Handles the racket*  *AH : Handles the shuttle* | 3 | 1 |
| 40 | Thread beads | 3 | 1 |
| 41 | Fixate paper while writing  *LH: Handles the pencil*  *AH: Fixates the paper* | 2 | 1 |
| 42 | Put on shoes | 1 | 1 |
