# Supplementary material for: Arm hand skilled performance in cerebral palsy: activity preferences and their movement components
Source: BMC Neurol. 2014 Mar 19;14:52. doi: 10.1186/1471-2377-14-52 (PMC4000003; doi:10.1186/1471-2377-14-52)
Supplement: Additional file 2: Table A2 — Overview of the most prominent movement components for the goals identified, pooled for all three age groups. [file 1471-2377-14-52-S2.doc]

***Additional File 2***

Table A2: Overview of the most prominent movement components for the goals identified, pooled for all three age groups.

|  | **Leading arm-hand** | | | | | | | | | |  | **Assisting arm-hand** | | | | | | | | | |
| --- | --- | --- | --- | --- | --- | --- | --- | --- | --- | --- | --- | --- | --- | --- | --- | --- | --- | --- | --- | --- | --- |
|  | **Indicated as most prominent** | | | | | | | | | |  | **Indicated as most prominent** | | | | | | | | | |
|  | **Positioning** | **Reach** | **Grasp** | **Hold** | **Release** | **Manipulate** | **Push/Pull/Shove** | **Displace** | **Fixate** | **Other** |  | **Positioning** | **Reach** | **Grasp** | **Hold** | **Release** | **Manipulate** | **Push/Pull/Shove** | **Displace** | **Fixate** | **Other** |
| Catch a ball |  |  |  | **X** |  |  |  |  |  |  |  |  |  |  | **X** |  |  |  |  |  |  |
| Comb one’s hair, make a ponytail |  |  |  |  |  | **X** |  |  |  |  |  |  |  |  |  |  | **X** |  |  |  |  |
| Cut a paper/cardboard template |  |  |  | **X** |  | **X** |  |  |  |  |  |  |  |  |  |  |  |  |  | **X** |  |
| Cut bread while eating |  |  |  | **X** |  |  |  |  |  |  |  |  |  |  | **X** |  |  |  |  |  |  |
| Cut meat while eating |  |  |  |  |  |  | **X** |  |  |  |  |  |  |  |  |  |  |  |  | **X** |  |
| Cut vegetables/fruit |  |  |  |  |  |  | **X** |  |  |  |  |  |  |  |  |  |  |  |  | **X** |  |
| Disassemble Lego toy building blocks |  |  |  |  |  |  | **X** |  |  |  |  |  |  |  |  |  |  | **X** |  |  |  |
| Displace a kettle and drain water for boiled potatoes |  |  |  | **X** |  |  |  |  |  | **X** |  |  |  |  | **X** |  |  |  |  |  | **X** |
| Displace a pile of plates |  |  |  | **X** |  |  |  | **X** |  |  |  |  |  |  | **X** |  |  |  | **X** |  |  |
| Displace boxes with toys |  |  |  | **X** |  |  |  | **X** |  |  |  |  |  |  | **X** |  |  |  | **X** |  |  |
| Displace more than 3 glasses at the same time |  |  |  | **X** |  |  |  | **X** |  |  |  |  |  |  | **X** |  |  |  | **X** |  |  |
| Do a handstand | **X** |  |  |  |  |  |  |  |  | **X** |  | **X** |  |  |  |  |  |  |  |  | **X** |
| Dress a doll |  |  |  |  |  | **X** |  |  |  |  |  |  |  | **X** |  | **X** |  |  |  |  |  |
| Dress a small toy doll |  |  |  |  |  | **X** |  |  |  |  |  |  |  |  | **X** |  |  |  |  |  |  |
| Dry oneself after bathing |  |  |  |  |  |  | **X** |  |  |  |  |  |  |  |  |  |  | **X** |  |  |  |
| Fasten a bracelet |  |  |  |  |  | **X** |  |  |  |  |  | **-** | **-** | **-** | **-** | **-** | **-** | **-** | **-** | **-** | **-** |
| Fixate paper while writing |  |  |  |  |  | **X** |  |  |  |  |  |  |  |  |  |  |  |  |  | **X** |  |
| Grasp and release objects |  |  | **X** |  |  |  |  |  |  |  |  | **-** | **-** | **-** | **-** | **-** | **-** | **-** | **-** | **-** | **-** |
| Handle a hockey stick |  |  |  | **X** |  |  | **X** |  |  |  |  |  |  |  | **X** |  |  | **X** |  |  |  |
| Hanging on the horizontal bar or swinging on the rings |  |  | **X** | **X** |  |  |  |  |  |  |  |  |  | **X** | **X** |  |  |  |  |  |  |
| Hold a jar while opening it |  |  | **X** |  |  |  |  |  |  | **X** |  |  |  |  |  |  |  |  |  | **X** |  |
| Hold a plate, mug, jar or bowl |  |  |  | **X** |  |  |  |  |  |  |  |  |  |  | **X** |  |  |  |  |  |  |
| Hold paper while cutting the paper | **X** |  |  |  |  | **X** |  |  |  |  |  | **X** |  |  |  |  |  |  |  | **X** |  |
| Hold the handlebars of a bicycle |  |  |  |  |  |  | **X** |  |  |  |  |  |  |  |  |  |  | **X** |  |  |  |
| Hold the handrail while climbing stairs | **-** | **-** | **-** | **-** | **-** | **-** | **-** | **-** | **-** | **-** |  |  |  | **X** |  |  |  | **X** |  |  |  |
|  | **Leading arm-hand** | | | | | | | | | |  | **Assisting arm-hand** | | | | | | | | | |
|  | **Indicated as most prominent** | | | | | | | | | |  | **Indicated as most prominent** | | | | | | | | | |
|  | **Positioning** | **Reach** | **Grasp** | **Hold** | **Release** | **Manipulate** | **Push/Pull/Shove** | **Displace** | **Fixate** | **Other** |  | **Positioning** | **Reach** | **Grasp** | **Hold** | **Release** | **Manipulate** | **Push/Pull/Shove** | **Displace** | **Fixate** | **Other** |
| Hold toys |  |  |  | **X** |  | **X** |  |  |  |  |  |  |  |  | **X** |  | **X** |  |  |  |  |
| Hold yourself while playing on the swings |  |  |  |  |  |  | **X** |  |  |  |  |  |  |  |  |  |  | **X** |  |  |  |
| Holding weights with two hands |  |  |  | **X** |  |  |  |  |  |  |  |  |  |  | **X** |  |  |  |  |  |  |
| Keep a tight grip while climbing |  |  | **X** |  |  |  | **X** |  |  |  |  |  |  | **X** |  |  |  | **X** |  |  |  |
| Lift a bicycle |  |  |  | **X** |  |  |  | **X** |  |  |  |  |  |  | **X** |  |  |  | **X** |  |  |
| Lift a pet |  |  |  | **X** |  |  |  |  |  |  |  |  |  |  | **X** |  |  |  |  |  |  |
| Make a sandwich |  |  |  |  |  | **X** | **X** |  |  |  |  |  |  |  |  |  |  |  |  | **X** |  |
| Making a knot to close a balloon |  |  |  |  |  | **X** |  |  |  |  |  |  |  |  |  |  | **X** |  |  |  |  |
| Open a bottle |  |  |  |  |  |  | **X** |  |  |  |  |  |  |  |  |  |  |  |  | **X** |  |
| Open/close a zipper |  |  |  |  |  | **X** | **X** |  |  |  |  |  |  |  |  |  |  |  |  | **X** |  |
| Open/close buttons |  |  |  |  |  | **X** |  |  |  |  |  |  |  |  |  |  |  |  |  | **X** |  |
| Operating machines |  |  |  |  |  |  | **X** |  |  |  |  | **-** | **-** | **-** | **-** | **-** | **-** | **-** | **-** | **-** | **-** |
| Pack a back |  |  |  |  |  |  |  | **X** |  |  |  |  |  |  |  |  |  |  | **X** |  |  |
| Peel potatoes |  |  |  |  |  |  | **X** |  |  |  |  |  |  |  | **X** |  | **X** |  |  |  |  |
| Pick up a tennis ball |  |  |  | **X** |  |  |  |  |  |  |  |  |  |  | **X** |  |  |  |  |  |  |
| Play badminton |  |  |  | **X** |  |  |  |  |  |  |  |  |  |  | **X** | **X** |  |  |  |  |  |
| Play on a game computer |  |  |  |  |  | **X** |  |  |  |  |  |  |  |  | **X** |  | **X** |  |  |  |  |
| Play rope-skipping |  |  |  | **X** |  |  |  |  |  | **X** |  |  |  |  | **X** |  |  |  |  |  | **X** |
| Play tennis | **X** |  |  | **X** |  |  |  |  |  |  |  |  |  |  |  | **X** |  |  |  |  |  |
| Play the guitar |  |  |  |  |  | **X** | **X** |  |  |  |  |  |  |  | **X** |  |  |  |  |  |  |
| Play the piano | **X** |  |  |  |  |  | **X** |  |  |  |  | **X** |  |  |  |  |  | **X** |  |  |  |
| Play with Duplo and K'nex |  |  | **X** |  |  | **X** |  |  |  |  |  |  |  | **X** |  |  | **X** |  |  |  |  |
| Pour a drink | **X** |  |  |  |  |  |  |  |  | **X** |  | **-** | **-** | **-** | **-** | **-** | **-** | **-** | **-** | **-** | **-** |
| Pulling on sailing ropes |  |  |  |  |  |  | **X** |  |  |  |  |  |  |  |  |  |  | **X** |  |  |  |
| Put on a coat | **X** |  |  |  |  |  |  |  |  |  |  | **X** |  |  |  |  |  |  |  |  |  |
| Put on a diving suit and diving shoes |  |  |  |  |  |  | **X** |  |  |  |  |  |  |  |  |  |  | **X** |  |  |  |
| Put on a watch |  |  |  |  |  | **X** |  |  |  |  |  | **-** | **-** | **-** | **-** | **-** | **-** | **-** | **-** | **-** | **-** |
| Put on a glove |  |  |  |  |  |  | **X** |  |  |  |  | **X** |  |  |  |  |  |  |  |  |  |
| Put on shin guards |  |  | **X** |  |  |  |  |  |  |  |  |  |  |  |  |  |  |  |  | **X** |  |
| Put on shoes |  |  |  |  |  |  | **X** |  |  |  |  |  |  |  |  |  |  | **X** |  |  |  |
| Put on socks |  |  |  |  |  |  | **X** |  |  |  |  |  |  |  |  |  |  | **X** |  |  |  |
| Put on/off a sweater |  |  |  |  |  |  | **X** |  |  |  |  |  |  |  |  |  |  | **X** |  |  |  |
| Put on/off trousers |  |  |  |  |  | **X** |  |  |  |  |  |  |  | **X** |  |  |  |  |  |  |  |
|  | **Leading arm-hand** | | | | | | | | | |  | **Assisting arm-hand** | | | | | | | | | |
|  | **Indicated as most prominent** | | | | | | | | | |  | **Indicated as most prominent** | | | | | | | | | |
|  | **Positioning** | **Reach** | **Grasp** | **Hold** | **Release** | **Manipulate** | **Push/Pull/Shove** | **Displace** | **Fixate** | **Other** |  | **Positioning** | **Reach** | **Grasp** | **Hold** | **Release** | **Manipulate** | **Push/Pull/Shove** | **Displace** | **Fixate** | **Other** |
| Put screws in |  |  |  |  |  | **X** |  |  |  |  |  |  |  |  | **X** |  |  |  |  |  |  |
| Shake hands with someone |  | **X** | **X** |  |  |  |  |  |  |  |  | **-** | **-** | **-** | **-** | **-** | **-** | **-** | **-** | **-** | **-** |
| Take a shower |  |  |  |  |  |  | **X** |  |  |  |  |  |  |  |  |  |  | **X** |  |  |  |
| Thread beads |  |  |  |  |  | **X** | **X** |  |  |  |  |  |  |  |  |  | **X** | **X** |  |  |  |
| Tie one’s shoelaces |  |  |  |  |  | **X** |  |  |  |  |  |  |  |  |  |  | **X** |  |  |  |  |
| Type on a keyboard |  |  |  |  |  |  | **X** |  |  |  |  |  |  |  |  |  |  | **X** |  |  |  |
| Use a mechanic mouse |  |  |  |  |  |  | **X** |  |  |  |  | **-** | **-** | **-** | **-** | **-** | **-** | **-** | **-** | **-** | **-** |
| Use cutlery while eating |  |  |  |  |  |  | **X** |  |  |  |  |  |  |  | **X** |  |  |  | **X** |  |  |
| Use keys |  |  |  |  |  | **X** |  |  |  | **X** |  | **-** | **-** | **-** | **-** | **-** | **-** | **-** | **-** | **-** | **-** |
| Use modelling clay |  |  | **X** |  | **X** | **X** |  |  |  |  |  |  |  | **X** |  | **X** | **X** |  |  |  |  |
| Use the handbrakes while bicycling |  |  |  |  |  |  | **X** |  |  |  |  |  |  |  |  |  |  | **X** |  |  |  |
| Wood crafting |  |  |  |  |  | **X** |  |  |  |  |  |  |  |  |  |  |  |  |  | **X** |  |
